# Conservation of functional domains and limited heterogeneity of HIV-1 reverse transcriptase gene following vertical transmission

**DOI:** 10.1186/1742-4690-2-36

**Published:** 2005-05-26

**Authors:** Vasudha Sundaravaradan, Tobias Hahn, Nafees Ahmad

**Affiliations:** 1 Department of Microbiology and Immunology, College of Medicine, The University of Arizona Health Sciences Center, Tucson, Arizona 85724, USA

## Abstract

**Background:**

The reverse transcriptase (RT) enzyme of human immunodeficiency virus type 1 (HIV-1) plays a crucial role in the life cycle of the virus by converting the single stranded RNA genome into double stranded DNA that integrates into the host chromosome. In addition, RT is also responsible for the generation of mutations throughout the viral genome, including in its own sequences and is thus responsible for the generation of quasi-species in HIV-1-infected individuals. We therefore characterized the molecular properties of RT, including the conservation of functional motifs, degree of genetic diversity, and evolutionary dynamics from five mother-infant pairs following vertical transmission.

**Results:**

The RT open reading frame was maintained with a frequency of 87.2% in five mother-infant pairs' sequences following vertical transmission. There was a low degree of viral heterogeneity and estimates of genetic diversity in mother-infant pairs' sequences. Both mothers and infants RT sequences were under positive selection pressure, as determined by the ratios of non-synonymous to synonymous substitutions. Phylogenetic analysis of 132 mother-infant RT sequences revealed distinct clusters for each mother-infant pair, suggesting that the epidemiologically linked mother-infant pairs were evolutionarily closer to each other as compared with epidemiologically unlinked mother-infant pairs. The functional domains of RT which are responsible for reverse transcription, DNA polymerization and RNase H activity were mostly conserved in the RT sequences analyzed in this study. Specifically, the active sites and domains required for primer binding, template binding, primer and template positioning and nucleotide recruitment were conserved in all mother-infant pairs' sequences.

**Conclusion:**

The maintenance of an intact RT open reading frame, conservation of functional domains for RT activity, preservation of several amino acid motifs in epidemiologically linked mother-infant pairs, and a low degree of genetic variability following vertical transmission is consistent with an indispensable role of RT in HIV-1 replication in infected mother-infant pairs.

## Background

The vertical transmission of human immunodeficiency virus type 1 (HIV-1) accounts for more than 90% of all HIV-1 infections in children. HIV-1 infected pregnant women can transmit the virus to their infants during all stages of their pregnancy, including prepartum (trans-placental passage), intrapartum (exposure of infants' skin and mucous membranes to contaminated maternal blood and vaginal secretions) and post-partum (via breast milk) at an estimated rate of 30% [1-4]. However, the rate of vertical transmission can be reduced by antiretroviral therapy during pregnancy. The risk of vertical transmission increases with several parameters, including advanced maternal disease status, low maternal CD4 cell count, high maternal viral load, recent infection of the mother, prolonged exposure of infant to ruptured membranes during parturition, and higher viral heterogeneity in the mother [5-8].

Viral heterogeneity is one of the classical means by which HIV-1 evades the host immune system. The heterogeneity of HIV-1 is attributed to the error-prone reverse transcriptase (RT) enzyme, which is responsible for converting the single stranded viral genomic RNA to double-stranded DNA that integrates into the host chromosome. As reverse transcription is the first step of the viral replication cycle [9], errors made at this stage ensures propagation of the erroneously copied genome to form the quasi-species of HIV-1 found in the infected individuals. These quasi-species infect other uninfected target cells and the cycle of error-prone reverse transcription continues. We have previously demonstrated that HIV-1 sequences from transmitting mothers (mothers who transmitted HIV-1 to their infants) were more heterogeneous compared with HIV-1 sequences from non-transmitting mothers (mothers who failed to transmit HIV-1 to their infants) [10]. This finding further suggests that the reverse transcription step that is responsible for generation of viral heterogeneity, may also play an important role in vertical transmission. The RT gene is unique in that it is also exposed to the same mutating effects of the RT enzyme as other part of the HIV-1 genome. Therefore, we sought to examine HIV-1 RT sequences from five infected mother-infant pairs following perinatal transmission.

The HIV-1 RT shows significant sequence and structural similarity to other viral reverse transcriptases as well as viral and bacterial RNA polymerases [11-13]. HIV-1 RT is a heterodimeric protein comprising of two subunits, 66 kDa and 51 kDa. It is encoded as a Gag-Pol precursor, Pr160^gag-pol^, which is cleaved by viral protease to yield the Gag protein and the viral polymerase which codes for RT [9,14]. The larger subunit (p66) of the heterodimer acts as an RNA-dependant DNA polymerase, a DNA-dependant DNA polymerase and has RNase H activity associated with the C-terminus [15,16], whereas the p51 subunit lacks the C-terminus RNase H activity, is folded differently from the p66 subunit and is thus inactive [17-20]. The p66 is folded to form a structure similar to a right hand with palm, finger and thumb subdomains [21-23] that are connected to the RNase H by the "connexion" subdomain [22,24,25]. Each domain has several secondary structural elements which are critical for primer binding, template binding [14,22,23,26,27] and nucleotide recruitment [28]. More specifically, the aspartate residues at position 110, 185 and 186 are believed to be the active sites of the polymerase and are located in the palm subdomain at the bottom of the DNA binding cleft [14,16,20,28,29]. Mutations in this subdomain and the active site abolish the enzymatic activity of HIV-1 RT [2,19,22,30-32] and alter viral replication, which may also affect HIV-1 mother-to-infant transmission.

In this study, we characterized the HIV-1 RT quasi-species from five mother-infant pairs following vertical transmission, including a mother with infected twin infants. We show that the open reading frame of the RT gene was highly conserved in the sequences from five mother-infant pairs. In addition, there was a low degree of heterogeneity and high conservation of functional domains essential for RT activity. These findings may be helpful in the understanding of the molecular mechanisms of HIV-1 vertical transmission.

## Results

### Patient population and sample collection

Blood samples were collected from five HIV-1-infected mother-infant pairs following perinatal transmission, including samples from a set of twins (IH1 and IH2) in the case of mother H. The demographic, clinical and laboratory findings on these mother-infant pairs are summarized in Table 1. The Human Subjects Committee of the University of Arizona, and the Institutional Review Board of the Children's Hospital Medical Centre, Cincinnati Ohio, approved this study. Written informed consent was obtained for participation in the study from mothers of infected mother-infant pairs.

**Table 1:** Demographic, Clinical, and Laboratory Parameters of HIV-1 Infected Mother-Infant Pairs

Patient	Age	Sex	CD4+ cells/mm3	Length of infection ^a^	Antiviral drug	Clinical Evaluation ^b^
MB	28 yr		509	11 mo	None	Asymptomatic
IB	4.75 mo	M	1942	4.75 mo	None	Asymptomatic, P1A
MC	23 yr		818	1 yr6 mo	None	Asymptomatic
IC	14 mo	F	772	14 mo	ZDV	Symptomatic AIDS;P2A,D1,3,F
MD	31 yr		480	2 yr6 mo	None	Asymptomatic
ID	28 mo	M	46	28 mo	ddC^c^	Symptomatic AIDS, P2AB,F; failed ZDV therapy
MF	23 yr		692	2 yr10 mo	None	Asymptomatic
IF	1 wk	M	2953	1 wk	ZDV	Asymptomatic,P1A
MH	33 yr		538	5 mo	None	Asymptomatic
IHT1	7 mo	F	3157	7 mo	ACTG152	Hepatosplenomeglay lymphadenopathy
IHT2	7 mo	F	2176	7 mo	ACTG152	Hepatosplenomegaly lymphadenopathy

M: mother; I: infant. ^a^Length of infection: The closest time of infection that we could document was the first positive HIV-1 serology date or the first visit of the patient to the AIDS treatment Center, where all the HIV-1 positive patients were referred to as soon as an HIV-1 test was positive. Therefore, these dates may not reflect the exact dates of infection.

^b ^Evaluation for infants is based on CDC criteria, ^c^ddC, Zalcitibine

### Phylogenetic analysis of RT sequences of mother-infant isolates

**Figure 1:**
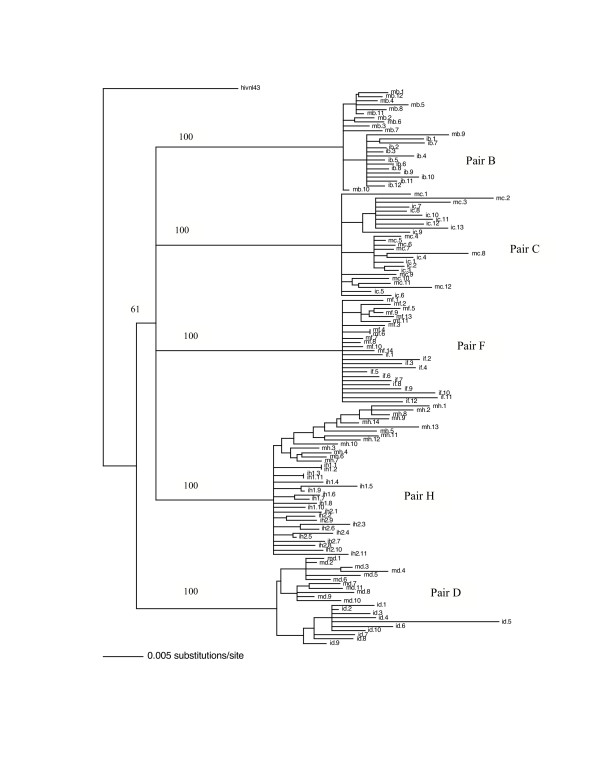
Phylogenetic analysis of HIV-1 RT of 132 RT sequences from five mother-infant pairs, including B, C, D, F and H. The neighbor-joining tree is based on the distance calculated between the nucleotide sequences from the five mother-infant pairs. Each terminal node represents one RT gene sequence. The numbers on the branch points indicate the percent occurrence of branches over 1,000 bootstrap resamplings of the data set. The sequences from each mother formed distinct clusters and are well discriminated and in confined subtrees, indicating that the variants from the same mother-infant pair are closer to each other than to other sequences and that there was no PCR cross-contamination. These data were strongly supported by the high bootstrap values indicated on the branch points.

We first performed multiple independent polymerase chain reaction (PCR) amplifications from peripheral mononuclear cells (PBMC) DNA of five mother-infant pairs and obtained 10 to 14 clones from each patient followed by nucleotide sequencing of these clones. We then performed the phylogenetic analysis by constructing a neighbor-joining tree of the 132 RT sequences from these mother-infant pairs, including the set of twins from mother H and the reference strain NL4-3, as shown in Figure 1. A model of evolution was optimized for the entire nucleotide sequence data set using the approach outlined by Huelsenbeck and Crandall [33]. The model of choice was incorporated into PAUP [34] to estimate a neighbor-joining tree and the tree was bootstrapped 1000 times to ensure fidelity. The phylogenetic tree demonstrated that the RT sequences from five mother-infant pairs were well discriminated in separate clusters and that the mother and infant sequences were generally separated in distinct subclusters. However, there was some intermingling between mother and infant sequences in pair C. Furthermore, the formation of separate subclusters of RT sequences from twins of mother H suggests that the there was probably compartmentalization of HIV-1 in the two fetuses causing independent evolution. We also compared our mother-infant pairs' RT sequences with the RT sequences of several clades present in the HIV databases and found that our RT sequences grouped with clade or subtype B sequences (not shown). The data on phylogenetic analysis indicate that the epidemiologically linked mother-infant sequences are closer to each other than epidemiologically unlinked sequences and that there was no PCR cross contamination. It is important to note that the mother-infant pairs grouped in the same subtree, even when some of the infants' ages were more than 2 to 3 years, suggesting that the epidemiological relationships are maintained in mother-infant pairs no matter how long the infection in the infants has progressed.

### Coding potential of RT gene sequences

**Figure 2:**
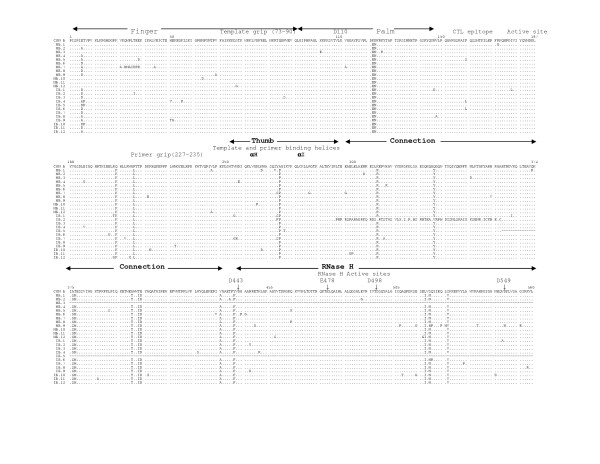
Multiple sequence alignment of deduced amino acids of HIV-1 reverse transcriptase (RT) gene from mother-infant pair B involved in vertical transmission. In the alignment, the top sequence is the consensus RT sequence of subtype or clade B (CON B) to which mother-infant pair-B RT sequences are aligned. In mother-infant pair B sequences, each line refers to a clone identified by a clone number with M referring to mothers and I referring to infants. The structural elements of RT are indicated above the alignment. Dots represent amino acid agreement with CON-B and substitutions are shown by single letter codes for the changed amino acid. Stop codons are shown as x and dashes represent gaps or truncated protein. Relevant amino acid motifs and domains essential for RT activity are shown by spanning arrowheads indicated above the alignment.

**Figure 3:**
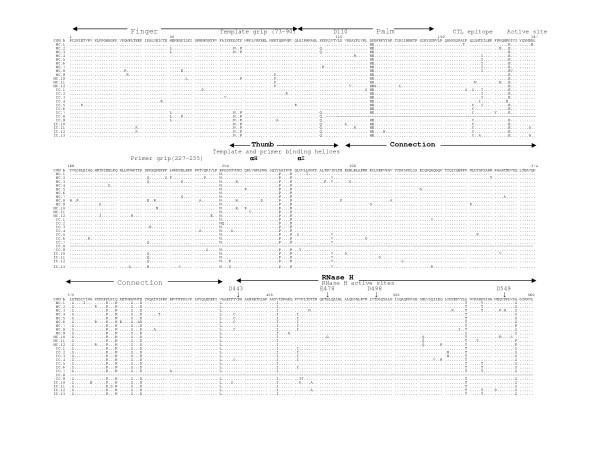
Multiple sequence alignment of deduced amino acids of HIV-1 reverse transcriptase (RT) gene from mother-infant pair C in reference to consensus subtype B (CON B) RT sequence. In the alignment, the top sequence is CON B RT sequence and the bottom sequences are mother-infant pair C sequences (M refers to mother sequences and I to sequences). The number of clones sequenced is represented with clone numbers. The structural elements of RT are indicated above the alignment. Dots represent amino acid agreement with CON-B and substitutions are shown by single letter codes for the changed amino acid. Stop codons are shown as x and dashes represent gaps or truncated protein. Spanning arrowheads indicated above the alignment shows relevant amino acid motifs and domains essential for RT function.

**Figure 4:**
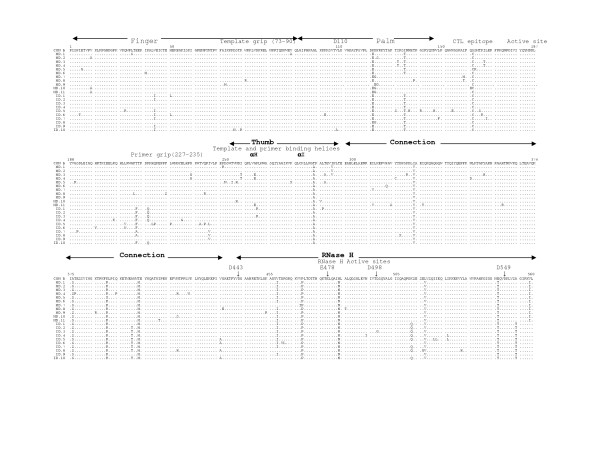
Multiple sequence alignment of deduced amino acids of HIV-1 reverse transcriptase (RT) gene from mother-infant pair D. The patient sequences are aligned in reference to consensus RT sequence of HIV-1 subtype or clade B (CON B) at the top. In the mother-infant pair sequences, each line refers to a clone identified by a clone number with M referring to mother and I to infants. The structural elements of RT are indicated above the alignment. Dots represent amino acid agreement with CON-B and substitutions are shown by single letter codes for the changed amino acid. Stop codons are shown as x and dashes represent gaps or truncated protein. Relevant amino acid motifs and domains essential for RT activity are shown by spanning arrowheads indicated above the alignment.

**Figure 5:**
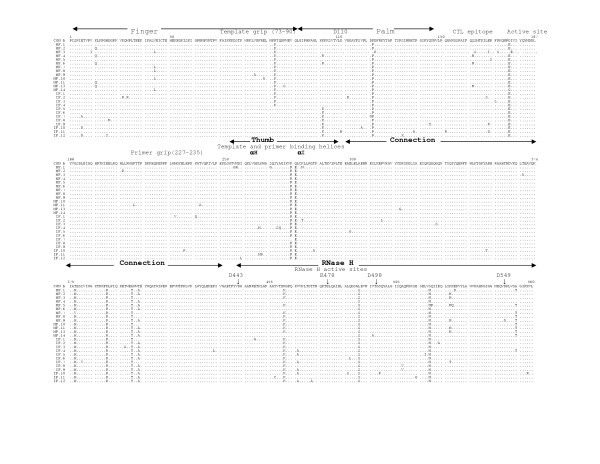
Multiple sequence alignment of deduced amino acids of HIV-1 reverse transcriptase gene from mother-infant pair F. In the alignment, the top sequence (CON B) is the consensus subtype B RT sequence and the bottom sequences are from mother-infant pair F sequences (M stands for mother sequences and I for infant sequences and the number of clones for mother and infant are indicated by clone number). The structural elements of RT are indicated above the alignment. Dots represent amino acid agreement with CON-B and substitutions are shown by single letter codes for the changed amino acid. Stop codons are shown as x and dashes represent gaps or truncated protein. Relevant amino acid motifs and domains essential for RT functions are shown by spanning arrowheads indicated above the alignment.

**Figure 6:**
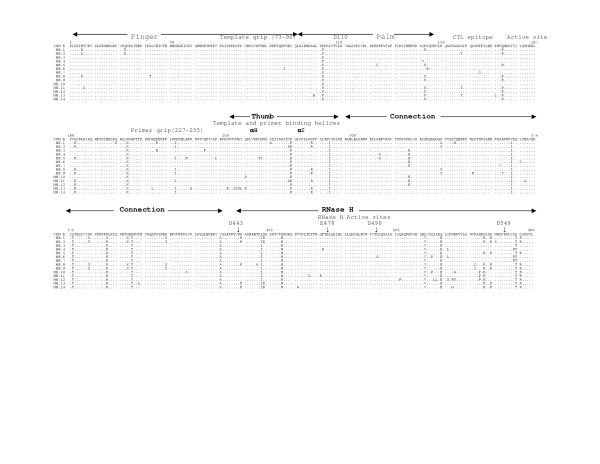
Multiple sequence alignment of deduced amino acids of HIV-1 reverse transcriptase (RT) gene from mother H, who had given birth to infected twins, H1 and H2 (alignment shown in Figure 7). In the mother H sequences, each line refers to a clone identified by a clone number with M referring to mother. The mother sequences are aligned in reference to consensus RT sequence of HIV-1 subtype or clade B (CON B) shown at the top. The structural elements of RT are indicated above the alignment. Dots represent amino acid agreement with CON-B and substitutions are shown by single letter codes for the changed amino acid. Stop codons are shown as x and dashes represent gaps or truncated protein. Spanning arrowheads indicated above the alignment shows relevant amino acid motifs and domains required for RT activity.

**Figure 7:**
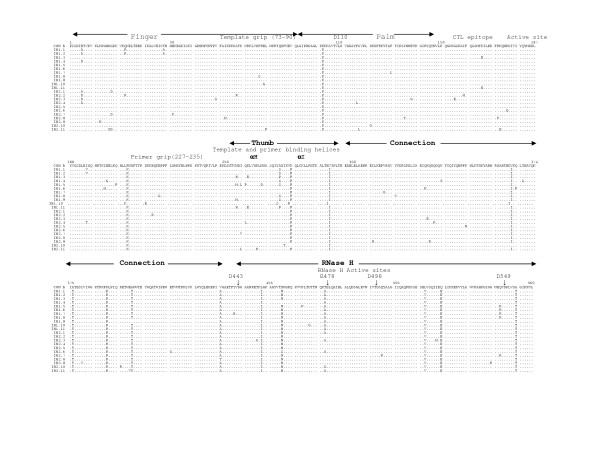
Multiple sequence alignment of deduced amino acids of HIV-1 reverse transcriptase gene (RT) from infected twin infants, H1 and H2 of mother H (alignment shown in Figure 6). In the alignment, the top sequence is the consensus subtype B RT sequence (CON B) and the bottom sequences are of infants H1 and H2 represented by I and clone numbers. Dots represent amino acid agreement with CON-B and substitutions are shown by single letter codes for the changed amino acid. Stop codons are shown as x and dashes represent gaps or truncated protein. Relevant amino acid motifs and domains essential for RT activity are shown by spanning arrowheads indicated above the alignment.

The multiple sequence alignments of the deduced amino acid sequences of HIV-1 RT genes from five mother-infant pairs, B, C, D, F, mother H and her twin infants IH1 and IH2 are shown in Figures 2, 3, 4, 5, 6, and 7, respectively. These sequences were aligned with consensus subtype B RT sequence (CON B). We found that 115 of the 132 sequences analyzed contained a complete RT open reading frame (ORF), with an 87.2% frequency of intact RT open reading frames thus indicating that the coding potential of the RT ORF was maintained in most of the sequences in 1680 bp sequenced. Moreover, the infected mothers' sequences showed a frequency of 85.5% of intact RT ORF while infants demonstrated a frequency of 88.5%. Several clones in mother-infant pair B and mother H were found to be defective due to a single nucleotide substitution, insertion or deletion resulting either in frame-shift or stop codons. The RT sequences also displayed patient and pair specific amino acid sequence patterns. Several amino acid motifs changes were observed in majority of the mother-infant pairs' sequences, including a glutamic acid (E) or proline (P) at position 122, an arginine (R) at 277, and a threonine (T) or serine (S) at 376 and 400.

### Variability of RT gene sequences in mother-infant isolates

The degree of genetic variability of RT sequences, measured as nucleotide and amino acid distances based on pairwise comparison (as described in Methods), was determined for the five mother-infant pairs' sequences, and is shown in Table 2. The nucleotide sequences of RT within mothers (mothers B, C, D, F and H) differed by 0.80, 1.76, 1.37, 1.21 and 2.90% (median values), respectively, ranging from 0 to 3.46%. The variability in the infant sets (infants B, C, D, F, H1 and H2) was similar to the mother sequences and differed by 0.80, 1.49, 1.37, 1.31, 0.64 and 1.24% (median values), respectively, ranging from 0 to 2.21%. Interestingly, the variability between epidemiologically linked mother and infant sets (pairs B, C, D, F and H) was also on the same order of 1.05, 1.7. 1.74, 1.22 and 1.45 (median values) respectively, ranging from 0 to 4.48%. Moreover, the amino acid sequence variability of RT within mothers (mothers B, C, D, F and H) differed by 1.26, 2.81, 1.98, 1.26 and 2.27% (median values), respectively, ranging from 0 to 5.51%. The variability within infants (infants B, C, D, F, H1 and H2) differed by 1.44, 2.35, 1.80, 1.62, 1.44 and 1.62% (median values), ranging from 0 to 4.57%, and between mother-infant pairs (pairs B, C, D, F and H) by 1.44, 2.90, 2.53, 1.44 and 2.17% (median values), ranging from 0 to 6.47%, respectively. We also determined sequence variability between epidemiologically unlinked individuals and found that the nucleotide distances ranged from 0 to 9.1% (median 5.4%) and amino acid from 0 to 12.4% (median 6.34%). The variability in general was lower between epidemiologically linked mother-infant pairs' sequences than epidemiologically unlinked individuals, suggesting that epidemiologically linked mother-infant pair sequences are closer to each other.

**Table 2:** Distances in the RT sequences within mother sets, within infant sets, and betweenmother-infant pairs

**Nucleotide distances**											
**Within mothers**				**Within infants**				**Between mother and infants**			

Pair	Min	Med	Max	Pair	Min	Med	Max	Pair	Min	Med	Max

MB	0.0	0.80	2.10	IB	0.0	0.80	1.30	B	0.0	1.05	2.05
MC	0.0	1.76	3.46	IC	0.0	1.49	2.17	C	0.0	1.70	3.26
MD	0.0	1.37	2.21	ID	0.0	1.37	2.21	D	0.0	1.74	4.48
MF	0.0	1.21	1.54	IF	0.0	1.31	2.93	F	0.0	1.22	2.08
MH	0.0	2.90	2.60	IH1	0.0	0.64	1.34	H	0.0	1.45	3.30
				IH2	0.0	1.24	1.75				
**Total**	**0.0**	**1.34**	**3.46**	**Total**	**0.0**	**1.48**	**2.21**	**Total**	**0.0**	**1.32**	**4.48**

											

**Amino acid distances**											

**Within mothers**				**Within infants**				**Between mother and infants**			

Pair	Min	Med	Max	Pair	Min	Med	Max	Pair	Min	Med	Max

MB	0.0	1.26	4.61	IB	0.0	1.44	2.72	B	0.0	1.44	4.57
MC	0.0	2.81	5.51	IC	0.0	2.35	4.01	C	0.0	2.90	5.51
MD	0.0	1.98	3.83	ID	0.0	1.80	4.57	D	0.0	2.53	6.47
MF	0.0	1.26	2.35	IF	0.0	1.62	3.09	F	0.0	1.44	3.09
MH	0.0	2.27	3.09	IH1	0.0	1.44	2.17	H	0.0	2.17	6.27
				IH2	0.0	1.62	2.72				
**Total**	**0.0**	**1.52**	**5.51**	**Total**	**0.0**	**1.42**	**4.57**	**Total**	**0.0**	**2.90**	**6.47**

M: mother; I: infant. Min: Minimum; Med: Median; Max: Maximum. Totals were calculated for all pairs together

We also investigated if the low variability of RT sequences seen in our mother-infant pair isolates is due to errors made by LA Taq polymerase used in our study. We did not find any errors made by the LA Taq polymerase when we used a known sequence of HIV-1 NL 4–3 for PCR amplification and DNA sequencing of the RT gene.

### Dynamics of HIV-1 RT gene evolution in mother-infant isolates

The maximum likelihood estimates and chi square tests performed by Modeltest 3.06 [35] suggested different models of evolution for each patient sample. The estimates of genetic diversity of RT sequences from the five mother-infant pairs were determined by using the Watterson model, assuming segregating sites and the Coalesce method assuming a constant population size. The estimates of genetic diversity shown as theta values (estimated as nucleotide substitutions per site per generation) are shown in Table 3. The levels of genetic diversity among infected mothers and infants, as estimated by Watterson method, ranged from 0.012 to 0.025 and 0.009 to 0.021, respectively. Similar results were obtained when the mother-infant pair populations were analyzed by the Coelesce method, with the values ranging from 0.020 to 0.058 in mothers and from 0.016 to 0.060 in infants. These data suggest that the mother and infant populations evolved very slowly and at similar rates. The differences observed in the estimates of genetic diversity between and mothers and infants sequences are not statistically significant.

**Table 3:** Estimates of genetic diversity of HIV-1 RT within mother sets and infant sets

**MOTHERS**				**INFANTS**			
	**N**	**θ_w_**	**θ_c_**			**θ_w_**	**θ_c_**

Mother B	12	0.015	0.038	Infant B	12	0.014	0.033
Mother C	12	0.025	0.058	Infant C	13	0.021	0.060
Mother D	11	0.017	0.042	Infant D	10	0.019	0.040
Mother F	14	0.012	0.029	Infant F	12	0.018	0.053
Mother H	14	0.020	0.020	Infant H1	11	0.009	0.016
				Infant H2	11	0.015	0.044
**Totals**	**63**	**0.018**	**0.037**		**69**	**0.016**	**0.041**

N – number of RT clones sequenced. θ_w _– genetic diversity as calculated by the Watterson method; θ_c _– genetic diversity as calculated by the Coelesce method. Totals were indicated as an average of all values.

### Rates of accumulation of nonsynonymous and synonymous substitutions

Selection pressure on the RT gene was estimated as a ratio of accumulation of non-synonymous to non-synonymous substitutions using the Nielsen and Yang model [36] as implemented in codeML [37]. Although there are several models to predict the rate of positive selection, most of these models assume that all sites in a sequence are under the same selection pressure with the same underlying dN/dS ratio [38]. As substitutions of critical regions of a protein can lead to deleterious mutations, it is unrealistic to make assumptions about equal degree of selection throughout the protein. In cases where positive selection is operating on proteins, it has been shown that only a limited number of amino acids may be responsible for adaptive evolution. In such a case, methods that estimate dN/dS ratios over an entire sequence may fail to detect positive selection even when it exists [39]. The codeML method uses the codon as a unit of evolution as opposed to a nucleotide, and thus allows us to estimate the percentage of positions that are being positively selected instead of averaging the rates of positive selection over the entire gene [39]. This method also provides the percentage of mutations that are conserved, neutral or positively selected based on dN/dS values of 0, 1 or > 1, respectively. The dN/dS values as well as the proportions of each site category estimated using the Nielsen and Yang model are shown in Table 4. As described in the methods, a dN/dS value of greater than 1 suggests positive selection. The percentage of the substitutions being positively selected is shown in column p3. Except for viral populations in infants C and F, all isolated populations were associated with dN/dS ratio >1, indicating positive selection. In case of infants C and F, there was no positive selection on the mutations and most of the substitutions were neutral. All mothers generally displayed a higher proportion of positively selected p3 sites as compared to the infants. Although the dN/dS values for infant H1 and H2 seem higher than mother H, closer observation shows that the percentage of sites undergoing positive selection is higher in the mother than in the twin infants. Table 4 shows that in mothers, over half the sites (66.6%) belong to the conserved p1 category, whereas the frequency of neutral and positively selected sites was equally distributed. This is in contrast to the viral population from the infants where the conserved site category (p1) had a frequency of only 36.5% and close to half the sites (55.7%) belongs to the neutral p2 category. Statistical analysis revealed that only the proportion of the neutral p2 category was significantly different between mothers' and infants' sequence viral populations (p < 0.05). This is signified by the case that all the sites in Infant F belonged to the p2 category. Higher proportion of p2 sites in infants have also been shown in the nef gene product in these same mother infant pairs [40]. The variable (positively selected) sites (p3) in the mothers' sequences were associated with dN/dS ratios that ranged from 2.34 to 8.9, with viral sequence populations from three mothers (MD, MF, MH) that displayed a dN/dS ratio of below three. This is in contrast to the infants' viral populations that were either associated with a dN/dS of below 1, indicating no directional selection (IC and IF), a dN/dS ratio between 3 and 4 (IB and ID) or a very high dN/dS ratio as found in the sequences isolated from the twins H1 and H2. This analysis showed that the RT gene in both the mothers and infants is under positive selection pressure.

**Table 4:** dN/dS values in HIV-1 RT sequences within mother sets and within infant sets.

**MOTHER**						**INFANT**					
	**N**	**P1**	**P2**	**P3**	**dN/dS**		**N**	**P1**	**P2**	**P3**	**dN/dS**

Mother B	12	53	18.8	27	8.9	Infant B	12	41	42	16	3.31
Mother C	12	55.5	43	1.3	6.09	Infant C	13	0	81.2	18.8	0.01
Mother D	11	70.6	5.7	23.6	2.52	Infant D	10	74.8	19.2	5.9	4.44
Mother F	14	81.7	7.8	10.4	2.67	Infant F	12	0	100	0	0.001
Mother H	14	72	0	27	2.34	Infant H1	11	47	50	2.8	14.04
						Infant H2	11	56	42	0.6	16.58
**Totals**		**66.5**	**15.1**	**18.4**	**4.50**		**69**	**36.5**	**55.7**	**7.8**	**6.39**

N – number of RT clones sequenced.; P1 = proportion of conserved codons as a percent; P2 = proportion of neutral codons as a percent; P3 = proportion of positively selected codons as a percent. dN/dS = ratio of synonymous to non-synonymous at P3 sites. Totals were calculates as an average of all values.

### Analysis of functional domains of RT in mother-infant pairs

HIV-1 RT is a heterodimeric protein comprising of two subunits, p66 and p51. The larger subunit of the heterodimer acts as an RNA-dependant DNA polymerase, a DNA-dependant DNA polymerase and an RNase H that is associated with the C-terminus [15,16]. The p66 is folded to form a structure similar to the right hand with palm, finger and thumb subdomains [21,23,32] that are connected to the RNase H by the "connexion" subdomain [22,24,25]. Each domain has several secondary structural elements, which are critical for primer binding, template binding [14,22,23,26,27,41] and nucleotide recruitment [28]. The active sites of the polymerase comprise of aspartic acid (D) residues at positions 110, 185 and 186, which are located in the palm subdomain at the bottom of the DNA binding cleft [22,23]. Mutations of these aspartic acid residues abrogates the polymerase activity of RT [22,23,29,32]. These aspartate residues of the RT active site were conserved within the five mother-infant pairs RT sequences. Furthermore, the D185 and D186 that form a part of an essential highly conserved YMDD [32,42,43] motif involved in binding to the 3'OH of the primer strand [14,26], were highly conserved in our mother-infant pairs' RT sequences (Figures 2 to 7). The amino acids at positions 73–90 that constitute the template grip required for positioning and binding the RT template near the active site of the RT [23], were also conserved in most of our RT sequences. The primer grip responsible for primer binding extends from amino acids 227 to 235 [22,23] and these amino acids were also conserved in the mother-infant RT sequences. The K263, K353 and R358 that form salt bridges with the phosphate groups [14,21,22,30,44] of the template and primer were found to be conserved in most of the RT sequences analyzed. The thumb subdomain of RT is comprised of two anti-parallel α helices, αH and αI, which bind to the opposite strand of dsDNA. The αH also directly inserts into the minor groove of the DNA [14,22,41]. Both these helices were generally conserved in our mother-infant RT sequences.

The connexion subdomain that links the RT to the RNase H and forms the floor of the template binding cleft [22,24,25,42], showed some substitutions, including V293I, A376S and A400T in our mother-infant RT sequences. Mutations at positions H361 and Y501 reduces RNase H activity [24]. Examination of the five mother-infant pairs' sequences revealed that these two positions were intact in all RT sequences (Figures 2 to 7). Furthermore, the RNase H active sites contain four acidic amino acid residues, D443, E478, D498 and D549 [22,24,25,41,42], which were highly conserved in our mother-infant pairs sequences. In addition, several substitutions were seen in regions of RT that are not known to have critical function. The relevance of these changes is not known.

### Mutations associated with anti-retroviral drug resistance

Several naturally occurring mutations in the *pol *gene in treatment-naïve patients have been reported [45,46], although most of these mutations are not seen in our RT gene sequences. In addition, these mutations found in treatment-naïve patients were usually seen in non-subtype B infections and our patient population was from subtype B infected individuals. These changes were usually in amino acids where the mutations did not actually confer nucleoside reverse transcriptase inhibitor (NRTI) drug resistance but were accessory mutations [46-48]. Several amino acid changes in RT seen in patients undergoing NRTI therapy are selected primarily with zidovudine (ZDV) treatment. These mutations referred to as thymidine analog mutations (TAMs) include M41L, D67N, K70R, L210N, T215Y/F and K219Q [47,49]. Since most of our infected mothers were treatment naïve but infants were actively on ZDV therapy or on other drugs (Table 1), we examined the RT sequences for ZDV resistant mutations (Figure 2). Several TAMs associated with drug resistance were observed in our infants C and D who were either on prolonged or failed ZDV therapy. These mutations included M41L in three clones from infant C and two clones in infant D, D67N and K70R in five clones from infant C, L210W in one clone from infant D and T215F in seven clones from infant D and K219Q in four clones from infant C and D. In addition, one clone from infant C had all the above mutations, indicating significant resistance to ZDV [46,50]. Although Mother C was not on any antiretroviral therapy two clones had TAMs at M41L and K219Q positions, suggesting that these mutations were naturally occurring. It is interesting to note that the infant of this mother yielded several clones with these two mutations. An R211K mutation known as an accessory mutation associated with NRTI resistance [46] was also observed in all mother-infant pair H clones.

### Immunologically relevant mutations in the CTL epitopes of RT

The cytotoxic T lymphocyte (CTL) responses have been shown to exert significant immune pressure during HIV-1 infection. Strong CTL responses are maintained in long-term nonprogressors and these responses correlate with decrease in viral load [51-55]. It has been shown that transmitting mothers have larger numbers of CTL escape variants as compared to non-transmitting mothers [56], emphasizing that CTL escape variants may become a part of circulating virus that influences vertical transmission [56,57]. Several regions in the RT gene have been shown to elicit strong CTL responses during HIV-1 infection. The CTL eptitope, TVLDVGDAY, between amino acid positions 107–115 , is highly conserved among known HIV-1 isolates [57]. This epitope contains the amino acid D110 which is part of the RT active site. This epitope was highly conserved in most of the mother-infant RT clones sequenced (Fig. 2).

Another motif, TAFTIPSI, between amino acid positions 128–135 is an HLA-B51 restricted epitope . This epitope is present in the palm region consisting of positions A129 and I135 as anchor residues [57]. This motif was mostly conserved in the RT sequences of the five mother-infant pairs analyzed. In addition, I135T mutation decreases CTL response but increasing concentration of mutant peptide re-establishes appropriate responses [57]. The I135T mutation was seen in several of our mother-infant pair's D sequences.

The next motif AIFQSSMTK from amino acid positions 158–166, comprising of I159, F160, K166 anchor residues and recognized by several HLA types, is conserved among known HIV-1 isolates and believed to be associated with vertical transmission [56,57]. Our mother-infant pairs' RT sequences showed conservation in this motif. Another CTL epitope YPGIKVRQL from positions 271–279 has been reported to be conserved in transmitting mothers and infants with several natural occurring variants [56], was also found to be conserved in our mother-infant pairs' RT sequences. In addition, a P272H mutation that causes significant loss of CTL response for this epitope [56] was not seen in any of the RT clones analyzed.

## Discussion

In this study, we show for the first time that reverse transcriptase open reading frames from five mother-infant pairs following perinatal transmission were maintained with a frequency of 87.2%. The functional domains required for reverse transcriptase activity in HIV-1 replication were highly conserved in most of the mother-infants sequences. We also demonstrate a low degree of sequence variability and estimates of genetic diversity for reverse transcriptase genes after mother-to-infant transmission. However, epidemiologically unlinked individual's sequences were more heterogeneous than epidemiologically linked mother-infant pair's sequences. Several motifs in reverse transcriptase responsible for primer and template binding and positioning and motifs involved in nucleotide recruitment were conserved in all mother-infant pairs' sequences. The data we show here are comparable to those of our previously analyzed conserved genes, including *gag*P17MA, *vif*, *vpr*, *tat *and *nef *[58-62]. Our findings suggest that an intact and functional reverse transcriptase open reading frame is essential for HIV-1 replication in mothers and their infants and low degree of viral heterogeneity is maintained following vertical transmission.

The RT open reading frame was maintained in 115 of the 132 sequences (1680 base pairs sequenced), whereas 17 sequences contained stop codons (Figure 2). The frequency of conservation in five mother-infant pairs was found to be 87.2%. The comparison of the RT sequences with those of other conserved genes from HIV-1 infected mother-infant pairs showed comparable frequency of conversation, including *gag *p17 (86.2%), *vif *(89.8%), *vpr *(92.1%), *tat *(90.9%), *nef *(86.2%) and *vpu *(90.12%). There was no significant correlation between the conservation of RT open reading frame and disease progression in mothers and infants [63-65]. Several amino acid motifs were found to be a signature characteristic of each mother-infant pair, even in older infants where infection has progressed for more than 3 years. Phylogenetic analysis of the RT sequences revealed that the five mother-infant pairs were well discriminated, separated and confined within subtrees (Fig. 1), indicating that the epidemiologically linked mother-infant pairs were closer to each other and that there was no PCR product cross-contamination [66,67]. In addition, most of the mother and infant sequences of the same pair formed separate subclusters, with little intermingling between sequences of mother and infant in some pairs. In some mother-infant pairs, minor variants of the mothers seem to be predominating in the infants, which was also seen in our previous V3 region analysis [68]. We also observed intermingling of sequences in mother-H and her infected twins, indicating that different mother's variants were transmitted to the twins. With respect to viral heterogeneity, there was a low degree of genetic variability in the RT sequences from mother-infant pairs estimated by several methods. Similar levels of genetic diversity were seen in other conserved genes of the same mother-infant pairs, including *gag*, *vif*, *vpr *and *tat *[59-61,69]. The low degree of genetic variability was observed in RT sequences of mothers and maintained in the infants following transmission, suggesting the essential nature of this gene in viral pathogenesis. It is important to note that the mother-infant pairs retained the same epidemiological relationship, even when some of the infant's age was more than 2 to 3 years. We believe this is an important finding that the epidemiological relationships as well as certain signature sequence motifs are maintained in mother-infant pairs or transmitter-recipient partners no matter how long the infection has progressed. This information may be critical in terms of vaccine development.

Examining the motifs of the deduced amino acid sequences of the RT gene from five mother-infant pairs, we found that the essential motifs required for RT activity were mostly conserved in our mother-infant pairs' sequences (Figure 2). The sites essential for primer binding, template binding, positioning of template and primer, which are located in α-Helix H and α-Helix I [22,23], were are all conserved in RT sequences (Figure 2). Specifically, the amino acids involved in recruitment of nucleotides during reverse transcription [28] were mostly conserved. The active sites of the polymerase are located in the palm subdomain at the bottom of the DNA binding cleft comprising of aspartic acid (D) residues at positions 110, 185 and 186 were conserved within the five mother-infant pairs' RT sequences. Furthermore, the D185 and D186 also form a part of an essential YMDD motif, which is highly conserved in known HIV-1 isolates [14,22,23,26,32,43], was also conserved in our mother-infant pairs' RT sequences analyzed.

Some of the amino acids of the connexion subdomain that are critical for RNase H activity and replication [9,24,25] are conserved in our RT sequences with several substitutions of compatible nature, including V293I, K358R, A376S, and A390T. These substitutions were located in the regions of the connexion that forms the base of the binding cleft. It is possible that such mutations in the binding cleft may change the size of the cleft and affect fidelity of the reverse transcriptase without affecting the active site. Further assessment also shows that our RT sequences harbor mutations in the connexion and RNase H subdomains that are not at the critical sites required for RT activity. The implications of these mutations can be studied by performing the biological characterization of these RT clones in the context of HIV-1 replication. It would be interesting to determine whether the degree of genetic variability and conservation of RT functional domains in non-transmitting mothers and compare their sequences with the data presented here. Nonetheless, the data described here suggest that functional domains of the RT enzyme, including reverse transcriptase, DNA polymerase and RNase H, were highly conserved in our five mother-infant pair sequences.

In terms of CTL epitopes in the RT gene, Wilson et al., have shown that the transmitting mothers have larger numbers of CTL escape variants as compared to non-transmitting mothers but the transmitted viruses carrying epitopes are not escape variants [56]. It is possible that the CTL responses studied are tissue specific and a representation of peripheral blood, and the virus and the CTL variants in the placenta, birth canal, and breast milk are different [70]. In addition, there is evidence suggesting that Nef and Pol specific CTLs found in breast milk showed no detectable responses in peripheral blood. Although several previously defined CTL motifs in the RT gene [56,57] were conserved in our RT sequences, other mutations that either abrogated or improved the CTL responses [56,57] were not seen in our sequences. The possibilities exist that the mutants observed in the CTL epitopes in our study may contribute to differential responses in a tissue specific manner and thus influence vertical transmission.

While antiretroviral treatment during pregnancy has reduced the risk of vertical transmission in the United States, HIV-1 infection in children, as a result of perinatal transmission, is still increasing rapidly in developing countries. There is a global need of better preventive strategies of HIV-1 vertical transmission. If we characterize the properties of the transmitted viruses, we can then develop interventions against the properties of the transmitted viruses. We have already shown that the minor genotypes with R5 phenotypes are transmitted from mothers to infants and are initially maintained in the infants with the same properties [71]. Additional data on the properties of HIV-1 from mothers and infants following perinatal transmission presented in this study may aid in a better understanding of the molecular mechanisms of vertical transmission and development of effective strategies for prevention and control of HIV-1 infection in children.

## Conclusion

We have demonstrated that an intact and functional RT gene was maintained in infected mother-infant pairs following perinatal transmission. In addition, there was a lower degree of viral heterogeneity and estimates of genetic diversity in epidemiologically linked mother-infant pairs compared with epidemiologically unlinked individuals. Several amino acid motifs were found as a signature sequences in each mother-infant pair. We also found that the functional motifs of RT responsible for reverse transcription, DNA polymerization and RNase H were highly conserved in mother-infant RT sequences. These findings support the notion that RT is essential for HIV-1 replication in mothers and their infected infants.

## Methods

### PCR amplification, cloning and nucleotide sequencing

Peripheral blood mononuclear cells (PBMCs) were isolated by a single step Ficoll-Hypaque procedure (Pharmacia-LKB) from whole blood samples of HIV-1-infected mother-infant pairs. DNA was isolated as described previously [68]. The HIV-1 RT gene was amplified by a two-step PCR method, first using outer primers RT1 (5 GTACAGTATTAGTAGGACCTACACCTGTC, 2470 to 2498, sense) and RT2 (5'AAAATCACTAGCCATTGCTCTCCAATTAC, 4307 to 4279, antisense) and then with nested primers RT3 (5'TGGAAGAAATCTGTTGACTCAGATTGG, 2507 to 2533, sense) and RT4, (5'TTCTCATGTTCTTGGGCCTTATCT, 4270 to 4244, antisense). Equal amounts of PBMC DNA (approximately 25 to 50 copies from each patient) as determined by end-point dilution was subjected to multiple (5 to 8) independent PCRs to obtain clones that were sequenced and analyzed. PCRs were performed according the modified procedure of Ahmad et al., [68] in a 25 μl reaction mixture containing 2.5 μl of 10X PCR buffer (100 mM Tris-HCL, pH 8.3, 100 mM KCl, 0.02% Tween 20), 2.5 mM MgCl_2_, 400 μM each of dATP, dCTP, dGTP and dTTP, 0.2 to 1.0 μM of each of outer primers, and 2.5 U of TaKaRa LA *Taq *polymerase (TaKaRa Biomedicals, Shiga, Japan). The reactions were carried out at 94°C for 30s, 45°C for 45s and 72°C for 3 min for 35 cycles, with the last cycle allowing for seven minutes of additional polymerization. After the first round of PCR, 4μl of the first-PCR product was used for nested PCR, using inner primers and same reagents at 94°C for 30s, 52°C for 45s and 72°C for 3 min for 35 cycles. We used negative control with each PCR amplification and a known HIV-1 DNA, pNL4-3, to assess errors generated by the LA *Taq *polymerase. To avoid contamination, all samples, reagents and PCR products were stored separately and dispensed in a separate room free of all DNA used in the lab. The PCR products were then visualized on a 1% agarose gel, excised ad extracted by using a QIAquick Gel Extraction kit (Qiagen Inc.). These DNAs were cloned into the TA cloning system (pCR 2.1-TOPO vector, Invitrogen Inc.) and transformed into chemically competent TOP10 cells (Invitrogen Inc.). The white colonies were screened for correct size inserts and 10 to 14 clones from each patient obtained from multiple independent PCRs were initially manually sequenced and then sequenced using University of Arizona Biotechnology Center automated system.

### Sequence analysis

The nucleotide sequences of HIV-1 RT gene (approximately 1680 bp) from five mother-infant pairs were analyzed with the Wisconsin package 10.1 version of the Genetics Computer group (GCG) and were translated to corresponding deduced amino acid sequences (560 amino acids). A multiple sequence alignment was performed for the nucleotide and amino acid sequences with a reference HIV-1 consensus clade or subtype B RT sequences with a gap-opening penalty of 10 and a gap extension penalty of 5 using Clustal X. The transitions were not weighted and the amino acids were scored using a BLOSUM matrix. A model of evolution was optimized for the entire nucleotide sequence data set using the approach outlined by Huelsenbeck and Crandall [33]. Likelihood scores for different models of evolution were calculated using PAUP [34] and a chi square test was performed by Modeltest 3.06 [34,35,40,72]. Using the Model test and Akaike Information Criterion [72], all the null hypotheses were rejected except a GTR+G model. The five rate categories were as follows: R (A-C) = 2.962, R (A-G) = 10.5176, R (A-T) 1.3663, R (C-G) = 0.6563, R (C-T) 12.5484, R (G-T) = 1. A gamma distribution with the shape parameter (α) of the distribution estimated from the data matrix via maximum likelihood was used to account for the rate of heterogeneity. This shape parameter α was = 0.7775. The model of choice was incorporated into PAUP [34] to estimate a neighbor-joining tree and the tree was bootstrapped 1000 times to ensure fidelity. Models to represent patterns of evolution of variants of each patient population were identified and were used to estimate corrected pairwise nucleotide distances using PAUP [34]. Amino acid distances were also estimated using the Jukes-Cantor model with the Wisconsin package 10.1 of GCG. The minimum, median and maximum nucleotide and amino acid distances for each patient and linked patient pairs were calculated from these data (Table 2). To analyze the evolutionary processes acting on the RT gene, we estimated the ratio of non-synonymous (dN) to synonymous (dS) substitutions by a maximum likelihood model using codeML, a part of the PAML [37] package. The Nielsen and Yang [36] model considers the codon instead of the nucleotide as the unit of evolution and incorporates three distinct categories of sites. Every mutation is three times more likely to cause a nonsynonymous than a synononymous substitution and codeML accounts for this bias. The first category p1 represents the sites that are conserved and invariable where dN/dS = 0. The second category p2 represents neutral sites where dN/dS = 1 and represents sites at which the dN and the dS are fixed at the same rate. The third category p3 represents sites that are under positive selection where the dN have a higher rate of fixation than dS proportionally and dN/dS >1. The dynamics of HIV-1 evolution was assessed using techniques of population genetics. In population genetics, genetic diversity is defined as θ = 2N_*ei*_μ, where N_*ei *_is the inbreeding effective population size and μ is the per nucleotide mutation rate per generation. The Watterson model based on segregating sites and the Kuhner model assuming constant population size were used to estimate differences in genetic diversity, using the program Coalesce,  which is part of the Lamarc software package. The tree files and the data matrixes from PAUP were used to estimate θ values as a measure of genetic diversity.

#### Nucleotide sequence accession numbers

The sequences have been submitted to GenBank with accession numbers AY560388 to AY560528.

## Competing interests

The author(s) declare that they have no competing interests.

## Authors' contributions

VS carried out the PCR, cloning, and sequencing. VS and TH performed the sequence analysis by computer programs. VS and NA participated in the experimental design, data interpretation and writing of the manuscript. All the authors read and approved the final manuscript.

## References

[B1] Lepage P, Van de Perre P, Carael M, Nsengumuremyi F, Nkurunziza J, Butzler JP, Sprecher S (1987). Postnatal transmission of HIV from mother to child. Lancet.

[B2] Lowe DM, Parmar V, Kemp SD, Larder BA (1991). Mutational analysis of two conserved sequence motifs in HIV-1 reverse transcriptase. FEBS Lett.

[B3] Weinbreck PLV, Denis F, Vidal B, Muvnier M, DeLumley I (1988). Postnatal transmission of HIV infection. Lancet.

[B4] Ziegler JB, Cooper DA, Johnson RO, Gold J (1985). Postnatal transmission of AIDS-associated retrovirus from mother to infant. Lancet.

[B5] Ahmad N (2000). Molecular mechanisms of human immunodeficiency virus type 1 mother-infant transmission. Adv Pharmacol.

[B6] Blanche S, Rouzioux C, Moscato ML, Veber F, Mayaux MJ, Jacomet C, Tricoire J, Deville A, Vial M, Firtion G (1989). A prospective study of infants born to women seropositive for human immunodeficiency virus type 1. HIV Infection in Newborns French Collaborative Study Group. N Engl J Med.

[B7] Mok JQ, Giaquinto C, De Rossi A, Grosch-Worner I, Ades AE, Peckham CS (1987). Infants born to mothers seropositive for human immunodeficiency virus. Preliminary findings from a multicentre European study. Lancet.

[B8] Ryder RW, Nsa W, Hassig SE, Behets F, Rayfield M, Ekungola B, Nelson AM, Mulenda U, Francis H, Mwandagalirwa K (1989). Perinatal transmission of the human immunodeficiency virus type 1 to infants of seropositive women in Zaire. N Engl J Med.

[B9] Gotte M, Li X, Wainberg MA (1999). HIV-1 reverse transcription: a brief overview focused on structure-function relationships among molecules involved in initiation of the reaction. Arch Biochem Biophys.

[B10] Matala E, Crandall KA, Baker RC, Ahmad N (2000). Limited heterogeneity of HIV type 1 in infected mothers correlates with lack of vertical transmission. AIDS Res Hum Retroviruses.

[B11] Larder BA, Kemp SD, Darby G (1987). Related functional domains in virus DNA polymerases. Embo J.

[B12] Kamer G, Argos P (1984). Primary structural comparison of RNA-dependent polymerases from plant, animal and bacterial viruses. Nucleic Acids Res.

[B13] Toh H, Hayashida H, Miyata T (1983). Sequence homology between retroviral reverse transcriptase and putative polymerases of hepatitis B virus and cauliflower mosaic virus. Nature.

[B14] Ding J, Hughes SH, Arnold E (1997). Protein-nucleic acid interactions and DNA conformation in a complex of human immunodeficiency virus type 1 reverse transcriptase with a double-stranded DNA template-primer. Biopolymers.

[B15] di Marzo Veronese F, Copeland TD, DeVico AL, Rahman R, Oroszlan S, Gallo RC, Sarngadharan MG (1986). Characterization of highly immunogenic p66/p51 as the reverse transcriptase of HTLV-III/LAV. Science.

[B16] Gotte M, Maier G, Gross HJ, Heumann H (1998). Localization of the active site of HIV-1 reverse transcriptase-associated RNase H domain on a DNA template using site-specific generated hydroxyl radicals. J Biol Chem.

[B17] Hizi A, McGill C, Hughes SH (1988). Expression of soluble, enzymatically active, human immunodeficiency virus reverse transcriptase in Escherichia coli and analysis of mutants. Proc Natl Acad Sci U S A.

[B18] Prasad VR, Goff SP (1989). Linker insertion mutagenesis of the human immunodeficiency virus reverse transcriptase expressed in bacteria: definition of the minimal polymerase domain. Proc Natl Acad Sci U S A.

[B19] Larder BA, Purifoy DJ, Powell KL, Darby G (1987). Site-specific mutagenesis of AIDS virus reverse transcriptase. Nature.

[B20] Le Grice SF, Naas T, Wohlgensinger B, Schatz O (1991). Subunit-selective mutagenesis indicates minimal polymerase activity in heterodimer-associated p51 HIV-1 reverse transcriptase. Embo J.

[B21] Boyer PL, Ferris AL, Clark P, Whitmer J, Frank P, Tantillo C, Arnold E, Hughes SH (1994). Mutational analysis of the fingers and palm subdomains of human immunodeficiency virus type-1 (HIV-1) reverse transcriptase. J Mol Biol.

[B22] Jacobo-Molina A, Ding J, Nanni RG, Clark AD, Lu X, Tantillo C, Williams RL, Kamer G, Ferris AL, Clark P (1993). Crystal structure of human immunodeficiency virus type 1 reverse transcriptase complexed with double-stranded DNA at 3.0 A resolution shows bent DNA. Proc Natl Acad Sci U S A.

[B23] Kohlstaedt LA, Wang J, Friedman JM, Rice PA, Steitz TA (1992). Crystal structure at 3.5 A resolution of HIV-1 reverse transcriptase complexed with an inhibitor. Science.

[B24] Julias JG, McWilliams MJ, Sarafianos SG, Alvord WG, Arnold E, Hughes SH (2003). Mutation of amino acids in the connection domain of human immunodeficiency virus type 1 reverse transcriptase that contact the template-primer affects RNase H activity. J Virol.

[B25] Julias JG, McWilliams MJ, Sarafianos SG, Arnold E, Hughes SH (2002). Mutations in the RNase H domain of HIV-1 reverse transcriptase affect the initiation of DNA synthesis and the specificity of RNase H cleavage in vivo. Proc Natl Acad Sci U S A.

[B26] Ding J, Jacobo-Molina A, Tantillo C, Lu X, Nanni RG, Arnold E (1994). Buried surface analysis of HIV-1 reverse transcriptase p66/p51 heterodimer and its interaction with dsDNA template/primer. J Mol Recognit.

[B27] Gao G, Orlova M, Georgiadis MM, Hendrickson WA, Goff SP (1997). Conferring RNA polymerase activity to a DNA polymerase: a single residue in reverse transcriptase controls substrate selection. Proc Natl Acad Sci U S A.

[B28] Harris D, Kaushik N, Pandey PK, Yadav PN, Pandey VN (1998). Functional analysis of amino acid residues constituting the dNTP binding pocket of HIV-1 reverse transcriptase. J Biol Chem.

[B29] Harris D, Yadav PN, Pandey VN (1998). Loss of polymerase activity due to Tyr to Phe substitution in the YMDD motif of human immunodeficiency virus type-1 reverse transcriptase is compensated by Met to Val substitution within the same motif. Biochemistry.

[B30] Boyer PL, Ferris AL, Hughes SH (1992). Cassette mutagenesis of the reverse transcriptase of human immunodeficiency virus type 1. J Virol.

[B31] Chao SF, Chan VL, Juranka P, Kaplan AH, Swanstrom R, Hutchison CA (1995). Mutational sensitivity patterns define critical residues in the palm subdomain of the reverse transcriptase of human immunodeficiency virus type 1. Nucleic Acids Res.

[B32] Mulky A, Sarafianos SG, Arnold E, Wu X, Kappes JC (2004). Subunit-specific analysis of the human immunodeficiency virus type 1 reverse transcriptase in vivo. J Virol.

[B33] Huelsenbeck JP, Crandall KA (1997). Phylogeny estimation and hypothesis testing using maximum likelihood. Annu Rev Ecol Sys.

[B34] Swofford DI (1999). *PAUP* *Phylogenetic analysis using parsimony and other methods *4.0.0b2*.

[B35] Posada D, Crandall KA (1998). MODELTEST: testing the model of DNA substitution. Bioinformatics.

[B36] Nielsen R, Yang Z (1998). Likelihood models for detecting positively selected amino acid sites and applications to the HIV-1 envelope gene. Genetics.

[B37] Yang Z (2000). Phylogenetic Analysis of Maximum Likelihood (PAML).

[B38] Nei M, Gojobori T (1986). Simple methods for estimating the numbers of synonymous and nonsynonymous nucleotide substitutions. Mol Biol Evol.

[B39] Zanotto PM, Kallas EG, de Souza RF, Holmes EC (1999). Genealogical evidence for positive selection in the nef gene of HIV-1. Genetics.

[B40] Hahn T, Ramakrishnan R, Ahmad N (2003). Evaluation of genetic diversity of human immunodeficiency virus type 1 NEF gene associated with vertical transmission. J Biomed Sci.

[B41] Jacobo-Molina A, Arnold E (1991). HIV reverse transcriptase structure-function relationships. Biochemistry.

[B42] Sarafianos SG, Das K, Tantillo C, Clark AD, Ding J, Whitcomb JM, Boyer PL, Hughes SH, Arnold E (2001). Crystal structure of HIV-1 reverse transcriptase in complex with a polypurine tract RNA:DNA. Embo J.

[B43] Huang H, Chopra R, Verdine GL, Harrison SC (1998). Structure of a covalently trapped catalytic complex of HIV-1 reverse transcriptase: implications for drug resistance. Science.

[B44] Boyer PL, Ding J, Arnold E, Hughes SH (1994). Subunit specificity of mutations that confer resistance to nonnucleoside inhibitors in human immunodeficiency virus type 1 reverse transcriptase. Antimicrob Agents Chemother.

[B45] Cornelissen M, van den Burg R, Zorgdrager F, Lukashov V, Goudsmit J (1997). pol gene diversity of five human immunodeficiency virus type 1 subtypes: evidence for naturally occurring mutations that contribute to drug resistance, limited recombination patterns, and common ancestry for subtypes B and D. J Virol.

[B46] Vergne L, Peeters M, Mpoudi-Ngole E, Bourgeois A, Liegeois F, Toure-Kane C, Mboup S, Mulanga-Kabeya C, Saman E, Jourdan J (2000). Genetic diversity of protease and reverse transcriptase sequences in non-subtype-B human immunodeficiency virus type 1 strains: evidence of many minor drug resistance mutations in treatment-naive patients. J Clin Microbiol.

[B47] Tantillo C, Ding J, Jacobo-Molina A, Nanni RG, Boyer PL, Hughes SH, Pauwels R, Andries K, Janssen PA, Arnold E (1994). Locations of anti-AIDS drug binding sites and resistance mutations in the three-dimensional structure of HIV-1 reverse transcriptase. Implications for mechanisms of drug inhibition and resistance. J Mol Biol.

[B48] Turner D, Brenner B, Wainberg MA (2004). Relationships among various nucleoside resistance-conferring mutations in the reverse transcriptase of HIV-1. J Antimicrob Chemother.

[B49] Turner D, Roldan A, Brenner B, Moisi D, Routy JP, Wainberg MA (2004). Variability in the PR and RT genes of HIV-1 isolated from recently infected subjects. Antivir Chem Chemother.

[B50] Shafer RW, Hsu P, Patick AK, Craig C, Brendel V (1999). Identification of biased amino acid substitution patterns in human immunodeficiency virus type 1 isolates from patients treated with protease inhibitors. J Virol.

[B51] Borrow P, Lewicki H, Wei X, Horwitz MS, Peffer N, Meyers H, Nelson JA, Gairin JE, Hahn BH, Oldstone MB, Shaw GM (1997). Antiviral pressure exerted by HIV-1-specific cytotoxic T lymphocytes (CTLs) during primary infection demonstrated by rapid selection of CTL escape virus. Nat Med.

[B52] Harrer T, Harrer E, Kalams SA, Barbosa P, Trocha A, Johnson RP, Elbeik T, Feinberg MB, Buchbinder SP, Walker BD (1996). Cytotoxic T lymphocytes in asymptomatic long-term nonprogressing HIV-1 infection. Breadth and specificity of the response and relation to in vivo viral quasispecies in a person with prolonged infection and low viral load. J Immunol.

[B53] Harrer T, Harrer E, Kalams SA, Elbeik T, Staprans SI, Feinberg MB, Cao Y, Ho DD, Yilma T, Caliendo AM (1996). Strong cytotoxic T cell and weak neutralizing antibody responses in a subset of persons with stable nonprogressing HIV type 1 infection. AIDS Res Hum Retroviruses.

[B54] Klein MR, van Baalen CA, Holwerda AM, Kerkhof Garde SR, Bende RJ, Keet IP, Eeftinck-Schattenkerk JK, Osterhaus AD, Schuitemaker H, Miedema F (1995). Kinetics of Gag-specific cytotoxic T lymphocyte responses during the clinical course of HIV-1 infection: a longitudinal analysis of rapid progressors and long-term asymptomatics. J Exp Med.

[B55] Rinaldo CR, Beltz LA, Huang XL, Gupta P, Fan Z, Torpey DJ (1995). Anti-HIV type 1 cytotoxic T lymphocyte effector activity and disease progression in the first 8 years of HIV type 1 infection of homosexual men. AIDS Res Hum Retroviruses.

[B56] Wilson CC, Brown RC, Korber BT, Wilkes BM, Ruhl DJ, Sakamoto D, Kunstman K, Luzuriaga K, Hanson IC, Widmayer SM, Wiznia A, Clapp S, Aman AJ, Koup RA, Wolinsky SM, Walker BD (1999). Frequent detection of escape from cytotoxic T-lymphocyte recognition in perinatal human immunodeficiency virus (HIV) type 1 transmission: the ariel project for the prevention of transmission of HIV from mother to infant. J Virol.

[B57] Menendez-Arias L, Mas A, Domingo E (1998). Cytotoxic T-lymphocyte responses to HIV-1 reverse transcriptase (review). Viral Immunol.

[B58] Hahn T, Ahmad N (2001). Genetic characterization of HIV type 1 gag p17 matrix genes in isolates from infected mothers lacking perinatal transmission. AIDS Res Hum Retroviruses.

[B59] Husain M, Hahn T, Yedavalli VR, Ahmad N (2001). Characterization of HIV type 1 tat sequences associated with perinatal transmission. AIDS Res Hum Retroviruses.

[B60] Yedavalli VR, Chappey C, Ahmad N (1998). Maintenance of an intact human immunodeficiency virus type 1 vpr gene following mother-to-infant transmission. J Virol.

[B61] Yedavalli VR, Chappey C, Matala E, Ahmad N (1998). Conservation of an intact vif gene of human immunodeficiency virus type 1 during maternal-fetal transmission. J Virol.

[B62] Yedavalli VR, Husain M, Horodner A, Ahmad N (2001). Molecular characterization of HIV type 1 vpu genes from mothers and infants after perinatal transmission. AIDS Res Hum Retroviruses.

[B63] Albert J, Wahlberg J, Leitner T, Escanilla D, Uhlen M (1994). Analysis of a rape case by direct sequencing of the human immunodeficiency virus type 1 pol and gag genes. J Virol.

[B64] Holmes EC, Zhang LQ, Simmonds P, Rogers AS, Brown AJ (1993). Molecular investigation of human immunodeficiency virus (HIV) infection in a patient of an HIV-infected surgeon. J Infect Dis.

[B65] Huang Y, Zhang L, Ho DD (1998). Characterization of gag and pol sequences from long-term survivors of human immunodeficiency virus type 1 infection. Virology.

[B66] Korber BT, Learn G, Mullins JI, Hahn BH, Wolinsky S (1995). Protecting HIV databases. Nature.

[B67] Wolinsky SM, Korber BT, Neumann AU, Daniels M, Kunstman KJ, Whetsell AJ, Furtado MR, Cao Y, Ho DD, Safrit JT (1996). Adaptive evolution of human immunodeficiency virus-type 1 during the natural course of infection. Science.

[B68] Ahmad N, Baroudy BM, Baker RC, Chappey C (1995). Genetic analysis of human immunodeficiency virus type 1 envelope V3 region isolates from mothers and infants after perinatal transmission. J Virol.

[B69] Hahn T, Matala E, Chappey C, Ahmad N (1999). Characterization of mother-infant HIV type 1 gag p17 sequences associated with perinatal transmission. AIDS Res Hum Retroviruses.

[B70] Sabbaj S, Edwards BH, Ghosh MK, Semrau K, Cheelo S, Thea DM, Kuhn L, Ritter GD, Mulligan MJ, Goepfert PA (2002). Human immunodeficiency virus-specific CD8(+) T cells in human breast milk. J Virol.

[B71] Matala E, Hahn T, Yedavalli VR, Ahmad N (2001). Biological characterization of HIV type 1 envelope V3 regions from mothers and infants associated with perinatal transmission. AIDS Res Hum Retroviruses.

[B72] Akaike H (1974). A new look at the statistical model identification. IEEE Trans Autom Contr.

